# Spectrum of MRI features of ganglion and synovial cysts

**DOI:** 10.1007/s13244-016-0463-z

**Published:** 2016-02-24

**Authors:** Nelson Neto, Pedro Nunnes

**Affiliations:** Radiology Department, Centro Hospitalar de Lisboa Ocidental, Lisbon, Portugal; Radiology Department, Hospital da Lapa, Dr. Campos Costa, Imagiologia Clínica, Oporto, Portugal

**Keywords:** Synovial cysts, Ganglion cysts, Facet joint, Extremities, MRI

## Abstract

Ganglion and synovial cysts occur mainly, but not necessarily, in association with osteoarthritis. Presentation varies widely, ranging from small, incidentally detected, asymptomatic lesions to giant ones that might be the source of symptoms, either due to their compressive effect on adjacent structures or due to complications, such as rupture. On magnetic resonance imaging they are typically presented as smooth, well-circumscribed, thin-walled, unilocular, and homogeneously T2-hyperintense lesions. An identifiable thin stalk communicating to the joint space is not infrequent. Nevertheless, depending on their age, anatomic location, and eventual complication, they might have many distinct appearances, including septae and internal debris, which the radiologist must be familiar with in order to accurately differentiate them from worrisome cystic-like lesions. With regard to this diversity, some illustrative cases are presented.

## Introduction

Ganglion cysts (GC) and synovial cysts (SC) are among the most frequently occuring benign cystic lesions in the joints. Degenerative joint disease is the main predisposing factor [1–6], but they might also be related to a number of other conditions such as trauma, rheumatoid arthritis, gout, and systemic lupus erythematosus [2–4].

Due to their strong similarities and their unclear pathogenesis, the scientific nomenclature associated with these lesions, labeled interchangeably in the literature, remains controversial. However, according to current evidence they are distinct, not only from an anatomopathological point of view, but also in their potential therapeutic approach [1]. By definition, SCs are herniations of the synovial membrane through the capsule of a joint filled by synovial fluid, which may or may not keep a communication with the joint [1–4]. They are thought to serve as drainage reservoirs for the excessive joint effusion in the setting of any arthropathy, escaping from its regular location through a one-way-valve mechanism into the area of least resistance [1, 2, 4]. Occasionally, such as in the hip and the knee, a pre-existing bursa may develop a communication with the joint and act exactly the same way, becoming enlarged [1]. This is the reason why the terms “SC” and “bursal enlargement” are often used interchangeably in the literature.

Contrary to SCs, GCs lack a synovial cell lining and are constituted by a dense collagenous capsule surrounding a mucopolysaccharide-rich gelatinous fluid [1–3, 6], similar to that of SC but at a higher concentration [1]. A developmental continuum between a true SC and GC of a synovial herniation followed by myxoid degeneration has even been theorized, but not confirmed [1, 6]. GCs may arise from the joint capsule, the ligaments, the tendon sheaths, the bursae, or the subchondral bone [1], being generally classified as juxta-articular, intra-articular or periosteal [2].

Except for the spine, where zygoapophyseal or facet joint cysts frequently cause radiculopathy, neurogenic claudication, sensory deficits and, to a lesser extent, motor deficits [5, 6], most SCs and GCs in the extremities are asymptomatic and incidentally found by imaging performed for other reasons. Symptoms mainly arise from a compressive effect in adjacent structures and less frequently from inflammatory changes related to complication by rupture, hemorrhage, and/or infection [2, 6].

Surgical excision of symptomatic, soft-tissue cystic lesions of this type, arthroscopic when possible, has been the advocated treatment so far, with satisfactory results. However, percutaneous image-guided procedures, including aspiration, with or without cyst rupture and/or steroid injection, are also effective alternatives that, despite the higher recurrence and failure rates, may avoid surgery without precluding it if warranted [3, 5–8]. Recent data suggest that the magnetic resonance imaging (MRI) features of SCs might help in the selection of patients who may benefit the most from nonsurgical intervention as a first treatment option, with T2-hyperintensity predicting a better outcome, probably due to the lower viscosity of their content making them easier to rupture [5]. The distinction between an SC and a GC may also help in orienting therapy toward correcting any coexisting arthropathy, frequent in SC, or in simply targeting the lesion itself by means of surgical excision for instance, which is more commonly required in GCs that are refractory to conservative therapy [1].

## Imaging approach

Ultrasound (US), as a low-cost, widely available modality, is the initial imaging method of choice for any palpable soft-tissue mass in the extremities, usually differentiating cystic from non-cystic ones [3, 8]. There is a level of evidence of A for a GC/SC in the hip, the knee, and the ankle/foot, and of C in the wrist, with an overall strength of recommendation of 3 [9].

MRI should be performed after US in doubtful cases. It has become the gold-standard modality in the characterization of periarticular cystic lesions, mainly due to its excellent soft-tissue contrast and extremely high diagnostic power [3]. Accurate distinction between benign and malignant soft-tissue masses, with estimated sensitivity and specificity of up to 95 %, has been reported for distal upper extremity GCs [10].

Regarding the optimal MRI quality in the study of this kind of lesion in the extremities, the smallest surface coil that covers the entire lesion should be chosen and an initial large field of view, including the contralateral side, followed by a smaller field of view targeted to the lesion, should be used. Imaging acquisition of at least two perpendicular planes is mandatory, usually including the following weighted-sequences: T1, proton-density (PD) or T2, with and without fat suppression (FS), or short inversion time inversion-recovery (STIR) [3].

The MRI protocol that better demonstrates facet joint SCs and GCs in the spine should include at least T2-weighted sequences acquired in both axial and sagittal planes [5, 6].

Delayed arthrography, either by means of radiography, computed tomography, or MRI, 1–2 h after intra-articular injection of water-soluble contrast agent, improves sensitivity of imaging in the demonstration of a cyst-joint communication, detectable in less than 50 % on standard US or MRI [1]. This is particularly valuable in the differential diagnosis between atypical GCs and cystic-like malignant tumors [11, 12].

## MRI features

As pointed out above, the radiologic distinction between an SC and a GC is frequently impossible, location being the most helpful criterion. As an example, while SC are very likely to occur around the knee and the hip, GC are most commonly found in the distal extremities, particularly in the wrist [1]. The distribution of GC and SC in the extremities varies widely, from adjacent to the articular surface to several centimeters distantly, extending to any direction [4]. On the other hand, facet joint SCs tend to present at an extradural location, usually close to the joint [6], and have an average axial size of around 10 mm [5, 6].

Regardless of their distinction, most SCs and GCs on MRI look like smooth, well-circumscribed, and homogeneous cystic masses of variable size, with giant ones mainly occurring in large joints such as the knee and the shoulder [2] and being more prone to cause erosion of the adjacent bone [4]. An identifiable thin stalk connecting to the joint space is not infrequent, although present in less than half of cases [1]. Cyst wall and septa, if present, should be thin [3, 6] and may present scattered hypointense calcific foci [5, 6]. According to their cystic nature, the internal content of non-complicated GCs and SCs is typically hypo- to isointense on T1-weighted images (WI) and homogenously hyperintense on T2, PD, and STIR-WI, the degree of this hyperintensity being believed to vary inversely with the protein content of the fluid [2, 5, 6]. Besides the simple unilocular cysts, a more complex but equally benign appearance with several septa, internal T2-hypointense debris, and even osseous loose bodies is not rare [2, 4]. Complication by hemorrhage or infection is responsible for wall thickening and internal heterogeneous hyperintensity on T1-WI and corresponding hypointense signals on T2-WI [2, 4, 5]. Rupture results in surrounding edema and fluid tracking [2, 4].

To summarize, the main features that any radiologist should be able to accurately describe are the precise location of the cyst and its relationship with the adjacent structures, so as to recognize signs of complication and rule out potentially worrisome solid components.

## Spine

### Facet joints

The great majority of SCs arising from facet joints occur in the lumbar spine, L4/L5 being the most affected level. These lesions are seldom reported in the cervical spine and are even rarer in the thoracic spine. The advent of imaging techniques has led to an increase in the detection of lumbar facet SCs, whose incidence is approximately 0.65 % [6].

Most patients are women in their sixties, usually presenting with chronic painful unilateral lumbar radiculopathy. Acute presentation of cauda equina syndrome secondary to intracystic hemorrhage has also been described [6].

**Fig. 1:**
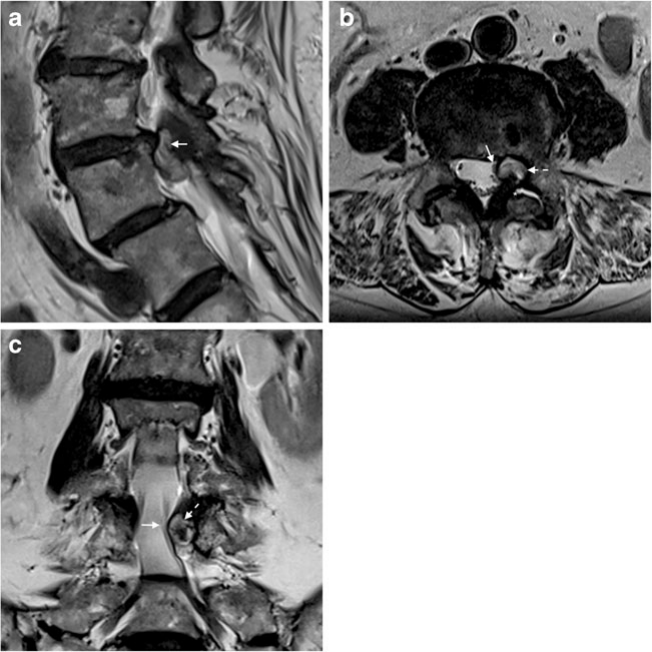
**a-c.** Lumbar facet synovial cyst in an 82-year-old woman presenting with subacute left lumbar radiculopathy and neurogenic claudication. Sagittal T2-weighted MRI (**a**) shows a slightly hyperintense cystic lesion posteriorly to the L3/L4 disc (arrow), as well as grade 1 degenerative spondylolisthesis at L4/L5. The axial view (**b**) clearly demonstrates the extradural location of the lesion (dashed arrow) arising from the left L3/L4 degenerated facet joint, which presents synovial effusion (asterisk). Note in both axial and coronal (**c**) views the displacement of the thecal sac and the left L4 nerve root (arrows) toward the right, due to compression by the cyst (dashed arrows). The partial T2-hypointensity, more evident in image c, might correspond to high-protein content or previous internal bleeding

**Fig. 2:**
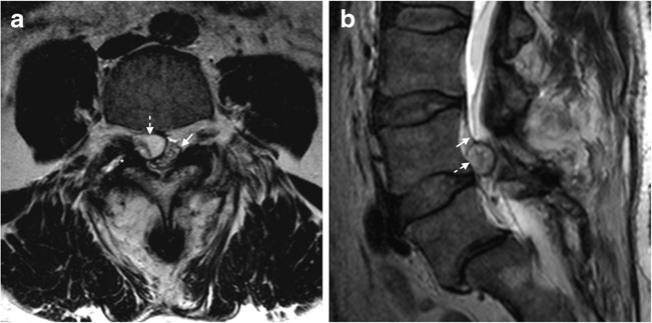
**a, b.** Lumbar facet synovial cyst in a 50-year-old man with a history of spinal surgery due to spondylolisthesis 20 years earlier, presenting with low back pain. Axial (**a**) and sagittal (**b**) T2-weighted images show a mildly hyperintense extradural rounded lesion (dashed arrows) arising from the right L4/L5 facet joint, which presents marked degenerative changes and fluid (asterisk). Note the compression of the thecal sac, displaced posteriorly (arrow in b) and to the left side (arrow in a). *Case courtesy of Dr. Carlos Casimiro. Neuroradiology Department, Centro Hospitalar de Lisboa Norte. Lisbon, Portugal*

The MRI features of both symptomatic facet SCs presented in Figs. 1 and 2, are similar to the typical pattern reported in the literature, which consists of rounded cystic lesions arising from the medial aspect of degenerated facet joints filled with synovial fluid, usually smaller than 22 mm. They tend to course with lateral recess stenosis and present dense adhesions to dura and nerve roots [6]. As previously mentioned, those with low-internal-signal intensity on the T2-WI are less likely to benefit from percutaneous rupture [5].

## Extremities

### Shoulder

Besides bursitis, most periarticular cysts in the shoulder are associated with labrocapsular or rotator cuff tears resulting in the passage of fluid from the joint to the pericapsular soft tissues. The main example, paralabral cysts, usually occurs in the setting of a superior or a posterosuperior labral tear, the cysts tending to extend into the suprascapular and the spinoglenoid notches, respectively, with resultant compression of the suprascapular nerve and subsequent denervation of the supra and infraspinatus muscles, or the infraspinatus muscle alone, if the site of compression is the spinoglenoid notch, distal to the branch to the supraspinatus [13, 14].

**Fig. 3:**
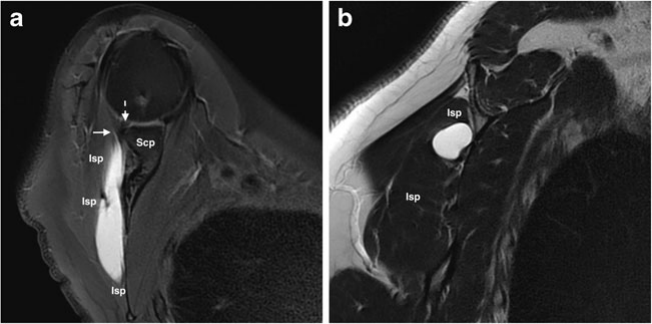
**a, b.** Intramuscular infraspinatus cyst in a 58-year-old woman with a known partial-thickness supraspinatus tear, presenting with exacerbated posterior right shoulder pain during elevation and external rotation. Axial FS T2-weighted MRI (**a**) shows a teardrop-shaped homogeneously hyperintense subaponeurotic intramuscular lesion along the posterior surface of the scapula. Note its thin extension toward the musculotendinous junction (arrow). The glenoid labrum (dashed arrow) seems preserved. A sagittal section (**b**) better demonstrates the location of this lesion within the infraspinatous muscle. Scp, scapula; Isp, infraspinatous

Far less common, acromioclavicular and intramuscular cysts are mainly but not necessarily associated with full- or partial-thickness rotator cuff tears, their presence improving the sensitivity and specificity of MRI detection of partial-thickness tears [14]. Intramuscular cysts of the shoulder are seldom-reported lesions, believed to be a result of fluid leakage through a defect in the musculotendinous junction of one of the rotator cuff muscles, dissecting within the fascial sheath or the muscle fibers. Due to their intramuscular location they are neither palpable at physical examination nor visible at surgery or arthroscopy [13]. Although typically small, large cysts with a long axis parallel to the length of the muscle, as the one illustrated in Fig. 3, occasionally occur. In case a rotator cuff tear is present, the cyst might occur either within the muscle of the torn tendon or within another adjacent rotator cuff muscle. Figure 3 shows an intramuscular infraspinatous cyst.

Regardless of the type of cyst depicted on MRI, considering the strong association, labral or rotator cuff tears must always be ruled out, as well as muscle atrophy.

### Wrist

**Fig. 4:**
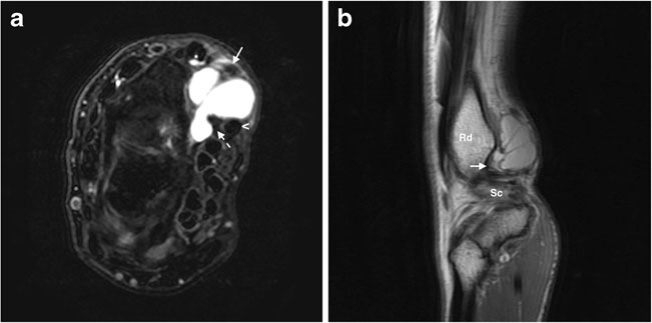
**a, b.** Ganglion cyst in the volar aspect of the wrist in a 55-year-old woman presenting with pain during volar flexion and paresthesia. Axial FS PD-weighted MRI (**a**) shows a smooth, multiloculated, homogeneously hyperintense lesion located deep and medial to the radial artery (arrow) and lateral to the flexor pollicis longus (dashed arrow) and the flexor carpi radialis (arrowhead) tendons. A tiny amount of fluid within the abductor pollicis longus and extensor pollicis brevis tendinous sheath (asterisk) is also present. The site of origin of the cyst is more evident on the sagittal T2-weighted MRI (**b**), which seems to originate more distally in the radioscaphoid interval (arrow). Rd, radius; Sc, scaphoid. *Case courtesy of Dr. Carlos Teiga. Radiology Department, Centro Hospitalar de Lisboa Central. Lisbon, Portugal*

With an estimated prevalence of 19 % in symptomatic patients having a MRI examination of the wrist and of 51 % in non-symptomatic ones [15], GCs are the most common soft tissue tumors of the distal upper extremity, the great majority occurring in the dorsal aspect of the wrist according to most studies [16]. There is a female predominance, usually affecting young patients in their twenties to their forties [16]. The scapholunate ligament in the dorsal aspect of the wrist is the most frequent site of origin. Less than 25 % of GCs of the wrist occur in the volar aspect, as the one presented in Fig. 4, most originating in the radioscaphoid-scapholunate interval, the scaphotrapezial, or the metacarpotrapezial joints [16].

Most GCs in the wrist are asymptomatic and easily diagnosed by physical examination, seen as a smooth nodular tumefaction of firm consistency on palpation of 1–2 cm that transilluminates, imaging remaining reserved for less obvious cases, especially those presenting with sensory and/or motor symptoms due to nerve compression. Nevertheless, spontaneous resolution occurs in up to 50 % of cases, and the main reason that patients seek medical evaluation is cosmetic concern, as symptoms are rarely significant [16].

As previously mentioned, US is the first-line imaging modality [9]. The GC is also the most accurate MRI-based diagnosis among distal, upper-extremity soft-tissue masses, with a sensitivity of 94.7 % and a specificity of 94.4 % [10].

US-guided aspiration is an effective procedure when treatment is required, reducing patient discomfort and the risk of damage to adjacent structures when compared to the non-guided technique [8], as well as avoiding the potential risks of surgery [16]. However, mainly due to its lower recurrence rate, surgical resection remains the gold-standard treatment option [8, 16], arthroscopy being a very promising alternative [16]. Steroid injection after aspiration does not seem to significantly improve the success rate of simple aspiration [16].

### Hip

**Fig. 5:**
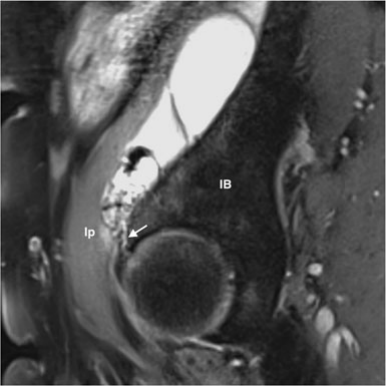
A giant synovial cyst incidentally found in the right hip of a 67-year-old woman during a routine computed tomography scan in the follow-up of a colorectal cancer in complete remission. For better characterization of the lesion, MRI was performed. Sagittal PD-WI shows a smooth, large multiloculated cyst, communicating with the joint space through a stalk (arrow). The lesion, probably corresponding to an enlarged iliopsoas bursa, displaces anteromedialy the iliopsoas muscle, and despite its close contact with the iliac bone, any erosion is seen. Ip, iliopsoas; IB, iliac bone

Cystic lesions around the hip are incidentally found in up to 26 % of asymptomatic patients during imaging studies [17]. After the knee, the hip is the second most-frequent joint where cystic lesions are more likely to consist of SCs rather than GCs, bursae being usually indistinguishable and also called SCs, as both are synovial-lined and may communicate with the joint, as previously mentioned [1, 17]. Among the fifteen normally occurring bursae around the hip, the iliopsoas bursa is the largest and the most constant, present bilaterally in 98 % of adults [17]. This bursa is located posteriorly to the musculotendinous junction of the iliopsoas muscle and communicates with the joint in 15 % of the normal population [1, 18]. As shown in Fig. 5, it may become extremely enlarged and present synovial hypertrophy, causing a condition known as iliopsoas bursitis, which is usually secondary to any disorder coursing with elevation of intra-articular pressure, such as osteoarthritis, and subsequent capsular rupture into the bursa or passage of fluid through a pre-existing connection [18]. The detection of iliopsoas bursitis is clinically relevant, as it constitutes an additional source of pain in patients with osteoarthritis [18]. To a lesser extent, but not rarely, other bursae such as the obturator and the trochanteric bursae might also become enlarged, with anatomical location being the distinctive feature [18].

Some other particular entities should be included in the differential diagnosis of a cystic lesion around the hip. Given their high prevalence, paralabral cysts deserve special mention. The mechanism of cyst formation is similar to that of paralabral cysts in the shoulder, with the passage of synovial tissue and/or fluid to the adjacent soft tissues through a labral tear, the majority occurring in the anterosuperior part of the acetabular labrum [17, 19]. As its name suggests, paralabral cysts are usually found close to the labrum. They communicate with the joint space and are typically multiloculated and small in size [19].

Besides its strong diagnostic power for the lesions described, US-guided drainage and steroid injection is extremely convenient for symptomatic relief of bursitis [9, 17, 18]. MRI is superior to US in the detection of smaller cysts and cyst-joint communications as well as associated disorders, such as acetabular labral tears and degenerative or inflammatory changes [17, 18].

### Knee

**Fig. 6:**
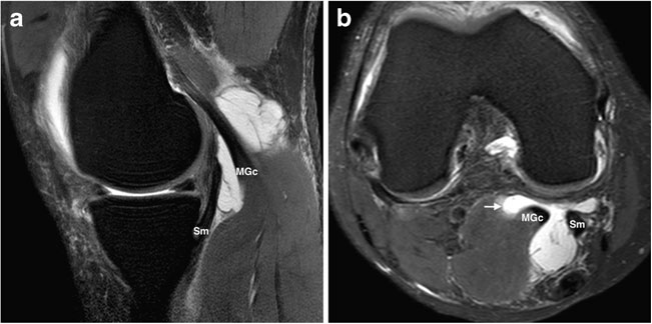
**a, b.** Baker’s cyst in a 33-year-old man presenting with nonspecific intermittent knee pain. Sagittal FS PD-weighted MRI (**a**) shows a hyperintense multiloculated fluid collection surrounding the medial gastrocnemius tendon. Its typical emergence between the medial head of the gastrocnemius muscle and the semimembranosus tendon is more evident on the axial view (**b**), as well as an intramuscular extension in its lateral aspect (arrow). MGc, medial gastrocnemius; Sm, semimembranosus

**Fig. 7:**
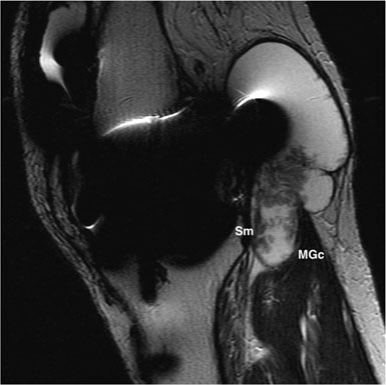
Heterogeneous popliteal cyst in an 89-year-old woman with known total knee arthroplasty presenting with a palpable mass. Sagittal T2-weighted MRI shows a few septa and hypointense internal debris in an otherwise common Baker’s cyst. Despite the severity of artifact due to metallic hardware, it is still possible to appreciate its relationship to the medial gastrocnemius and the semimembranosus tendons. Absence of enhancement after gadolinium intravenous administration was confirmed in the same study (not shown). MGc, medial gastrocnemius; Sm, semimembranosus

**Fig. 8:**
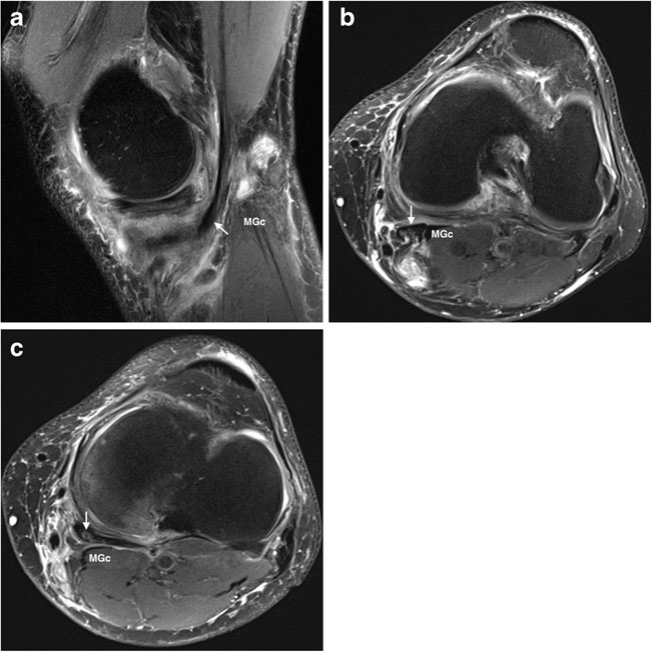
**a-c.** Ruptured Baker’s cyst in a 62-year-old man presenting with acute pain in the popliteal fossa and the medial side of the left leg after a run. Sagittal FS PD-weighted MRI (**a**) shows diffuse hypodermic edema and a popliteal cyst with irregular and undefined margins in close relation to the medial gastrocnemius muscle and the semimembranosus tendon (arrow). Axial FS PD-WI MRI along the proximal-to-distal axis (**b** and **c**) better demonstrates the intrasubstance edema of the semimembranosus tendon (arrows) so as to show the fluid tracking along the medial side of the leg, adjacent to the medial gastrocnemius muscle and the pes anserinus. MGc, medial gastrocnemius

The joint most commonly affected by SCs is the knee. Although popliteal or Baker’s cysts are not true SCs, in practical terms, they are considered similar for the same reason described above with regard to an iliopsoas bursa. Present in up to 38 % of knees imaged by MR, they consist of an enlarged gastrocnemius-semimembranosus bursa, which in more than 50 % of the general population normally communicates with the joint space through a synovial protrusion that follows the path of least resistance in the posteromedial aspect of the joint capsule [1]. Located between the tendons of the medial gastrocnemius and the semimembranosus muscles, regardless of its classical inferomedial extension, Baker’s cysts might follow any direction and even dissect intramuscularly [1, 2], as shown in Fig. 6. The larger they are the more they are prone to present with internal heterogeneous content, as shown in Fig. 7, so as to complicate by rupture with resultant inflammation of the surrounding soft tissues, as illustrated in Fig. 8.

Although far less common than a Baker’s cyst, SCs may arise from other locations around the knee, such as the tibiofibular joint, which communicates with the knee joint in 10 % of adults [2]. An important implication of proximal tibiofibular joint GCs is their potential to produce nerve impingement, with or without dissection. GCs arising from the anterior portion of the tibiofibular joint tend to affect the superficial peroneal nerve, while tibial intraneural ganglia are derived from the posterior portion of the same joint, inside the articular branch of the tibial nerve [20]. The preoperative recognition of these anatomical landmarks and the distinction between extra and intraneural cysts are crucial for the treatment outcome [20]. Contrary to extraneural GC, which tend to present with a globular appearance, intraneural cysts are usually tubular lesions following the expected course of a nerve branch [20].

**Fig. 9:**
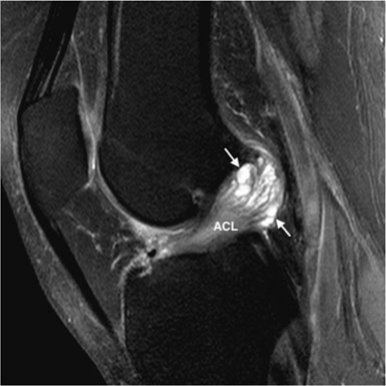
Anterior cruciate ligament ganglion cyst incidentally found in a 58-year-old woman during an MRI scan performed in the setting of a knee sprain. Sagittal FS PD-WI shows an enlarged anterior cruciate ligament due to a multiloculated cystic lesion (arrows) embedded within its fibers. ACL, anterior cruciate ligament

**Fig. 10:**
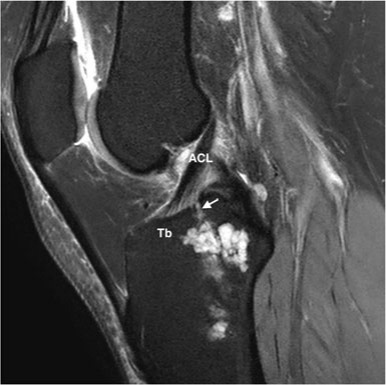
Intraosseous ganglion cyst of the tibia incidentally depicted in a 40-year-old man who underwent an MRI scan due to intermittent, subacute non-specific knee pain. Sagittal FS PD-WI shows a metaepiphyseal, large, multiloculated cystic lesion of the tibia, which communicates with the articular surface through a thin stalk (arrow) extending into the interspinous region, close to the anterior cruciate ligament tibial insertion. Tb, tibia; ACL, anterior cruciate ligament

Besides the general risk factors for the development of intra- and periarticular cysts described for other joints, such as osteoarthritis, some other knee-specific disorders include meniscal and cruciate ligament lesions [2]. In fact, although relatively infrequent, the knee joint is also a known location for GCs, particularly intra-articular ones. They might be found adjacent or within the cruciate ligaments [1, 2], most commonly in the anterior cruciate ligament [2], as illustrated in Fig. 9. Intraosseous GCs typically occur in the epiphyseal-metaphyseal region of long bones, the proximal tibia being the most frequently reported location within the knee [1, 2]. These cysts might be large, multiloculated lesions communicating with the joint space, as the one shown in Fig. 10.

Although MRI is the gold-standard technique in characterizing cystic lesions in the knee [3], US is also highly accurate and provides guidance for percutaneous therapies [9].

## Conclusion

SCs and GCs occur frequently but not necessarily in association with osteoarthritis. The knee and the wrist are the most commonly involved joints, but their occurrence in other sites such as the facet joints, the shoulder, and the hip is not as rare as traditionally believed. Their typical appearance on MRI consists of a smooth, well-circumscribed, thin-walled, homogeneous cystic lesion, not infrequently with an identifiable pedicle connecting to the joint. Nevertheless, a more complex appearance with thin septae and internal T2-hypointense debris should not be misinterpreted, neither as complication signs nor as malignant-like ones. Wall thickening and irregularity, internal heterogeneous T1-hyperintense serohematic content, and surrounding edema suggest acute complication.
