# Erratum to: Spectrum of MRI features of ganglion and synovial cysts

**DOI:** 10.1007/s13244-016-0488-3

**Published:** 2016-04-08

**Authors:** Nelson Neto, Pedro Nunnes

**Affiliations:** Radiology Department, Centro Hospitalar de Lisboa Ocidental, Lisbon, Portugal; Radiology Department, Hospital da Lapa, Porto, Portugal

**Erratum to: Insights Imaging**

**DOI 10.1007/s13244-016-0463-z**

Due to a transcription error, the teaching points were mistakenly excluded from this article.

Please find the teaching points below:

*Teaching Points*

• *Ganglion and synovial cysts are frequently associated with osteoarthritis.*

• *A smooth, thin-walled, homogeneous cyst with a stalk connecting to the joint is typical.*

• *Thin septa and internal debris should not be misinterpreted as worrisome findings.*

• *Wall thickening and irregularity, hematic content and surrounding fluid suggest acute complication.*

